# Current status of knowledge, attitudes, and practices of barbers regarding transmission and prevention of hepatitis B and C virus in the north-west part of Bangladesh: A cross-sectional study in 2020

**DOI:** 10.1016/j.puhip.2021.100124

**Published:** 2021-04-15

**Authors:** Md Abdul Mumit Sarkar, Madhusudan Saha, Mohammad Naymul Hasan, Birendra Nath Saha, Avijit Das

**Affiliations:** a Department of Gastroenterology, Rajshahi Medical College, Rajshahi, Bangladesh; b Department of Gastroenterology, North East Medical College, Sylhet, Bangladesh; c Department of Gastroenterology, Shaheed Ziaur Rahman Medical College, Bogura, Bangladesh; d Department of Gastroenterology, Shaheed Tajuddin Ahmad Medical College, Gazipur, Bangladesh; e Department of Microbiology, North East Medical College, Sylhet, Bangladesh

**Keywords:** Knowledge, Attitudes, Practices, Barbers, HBV, HCV

## Abstract

**Objectives:**

The objective of this study was to assess knowledge, attitudes, and preventative practices among barbers regarding Hepatitis B (HBV) and Hepatitis C (HCV) viral infection in Rajshahi city of Bangladesh.

**Study design:**

This was a prospective observational study.

**Methods:**

This study was conducted from January to June 2020. A questionnaire was adapted from existing knowledge attitude and practice surveys regarding HBV and HCV infection. Data were collected from barbers by face to face interview by trained interviewers. A knowledge score was calculated by giving one point for each correct response to the knowledge-related items, with a maximum score of 17. Associations between knowledge and patient characteristics were assessed by Welch’s *t*-test.

**Results:**

A total of 403 barbers were enrolled with a mean age of 29.2 ​± ​7.4 years. Of participants, the majority of barbers were either illiterate or had education up to primary school (232, 57.5%). 41.2%, 33.7%, and 25.1% barbers knew that HBV and HCV could be transmitted through sexual route, blood transfusion, and shaving instruments respectively. A lower knowledge level was associated with a lower level of education (P ​< ​0.001). Most of the participants agreed that it was essential to have periodic screening tests for HBV and HCV infection (318, 78.9%). Only 12.2% of the barbers were vaccinated against HBV. 57.1% of the barbers cleaned their instruments with disinfectant between clients. Most of them used a new blade on new clients (401, 99.5%) and disposed of the used blades into the regular garbage system (371,92.1%)

**Conclusions:**

Poor knowledge regarding transmission and prevention of HBV and HCV was found among barbers which could be one of the major causes of transmission of those infections in the community. The concerned authority should pay attention to the problems and can take specific measures to increase awareness of the barbers to prevent transmission of HBV and HCV from the barber’s shop.

## Highlights

• Majority of the barbers didn’t know that the viruses can be transmitted through their shaving instruments.
• Most of the barbers are not vaccinated against Hepatitis B virus.
• A large proportion of the barbers didn’t clean instruments with disinfectant between clients.
• Most of the barbers disposed of the used blades into the regular garbage system.

## 1 Introduction

Hepatitis B virus (HBV) and Hepatitis C virus (HCV) are the hepatotropic viruses leading to chronic hepatitis [1]. Approximately 248 million people and 71 million people are chronically infected with HBV and HCV worldwide, respectively, with a wide geographical variation [2,3]. In Bangladesh, the prevalence of HBV and HCV is 5.4% and 0.88%, respectively [4,5]. Infections with the viruses are also the leading causes of liver cirrhosis and hepatocellular carcinoma in the world and Bangladesh [6,7]. A large proportion of the patients infected with HBV and HCV remains asymptomatic until they present with features of decompensation [8]. Cirrhosis of the liver causes significant morbidity and mortality [9] and decreased quality of life even in uncomplicated patients [10]. Moreover, liver cirrhosis patients need drug treatment, regular follow-up with several investigations, and hospital admission for complications which cause an increase in the expenditure of direct and indirect healthcare costs [11]. Thus infection with HBV and HCV remains an important global public health problem.

Both HBV and HCV are mainly transmitted through parenteral and sexual routes though approximately 26% of patients with acute hepatitis B and 15% of patients with acute hepatitis C infection have no recognized risk exposure for those infections [12]. In a search for those unknown sporadic sources of infection, reuse of needles for ear and nose piercing, reuse of injections, injecting drug users, tattooing, unsterilized dental and surgical instruments, haircutting, and shaving by barbers are reported to be major risk factors for HBV and HCV infection in several studies [[13], [14], [15], [16]]. Moreover, HBV and HCV infections have also been implicated as an occupational hazard of the barbers’ trade in developing countries [5,[17], [18], [19], [20], [21]]. In Turkey, 39.8% of barbers were found to be HBV positive, and many were infected during the period of their employment [22]. Many of the barbers reuse razors and scissors on multiple clients without proper disinfection, indicating their low level of awareness about viral hepatitis and the risk of transmission of infectious agents [23,24]. Moreover, HCV remains alive for a long period of time on Potash Alum stone being used by barbers on facial shaving cuts and might play a definite role in HCV transmission [25]. The majority of the barbers in resource-poor countries are not vaccinated against HBV, which makes them vulnerable to the infection acquired by accidental exposure to their customers’ blood and body fluids during shaving and haircutting [[26], [27], [28]]. So it is important to know how barbers perceive the risks concerning the prevention of transmission of infection between themselves and customers so that appropriate prevention and intervention strategies could be taken to reduce the risk of HBV and HCV transmission from barber’s shop. Data on the knowledge, attitudes, and practices of barbers about HBV and HCV in Bangladesh, one of the world’s densely populated countries, are lacking. Hence the study was aimed to investigate the knowledge, attitudes, and practices (KAP) regarding hepatitis B and C viral infections among barbers.

## 2 Methods

### 2.1 Study location and period

The study was conducted at Rajshahi city from January 2020 to June 2020. Rajshahi is a metropolitan city and a major commercial and educational center in Bangladesh. It is also the administrative seat of the eponymous division and district. The city is situated in the north-west part of the country with an area of 97 ​km^2^ and a total population of more than 4.5 million [29].

### 2.2 Survey questionnaire development

As there is no Bangla validated KAP survey questionnaire for HBV and HCV, the survey questionnaire used in the present study was derived from multiple existing KAP surveys related to HBV and HCV in different countries [[26], [27], [28],^30^]. Candidate questions from existing surveys were selected and reviewed by an interdisciplinary team consisting of gastroenterologists, epidemiologists, physicians, and lay people. From this pool of questions, items were chosen for inclusion in the KAP survey based on cultural relevance, clarity of question, and unique content. The draft KAP survey questionnaire was first translated from English to Bangla and then back-translated from Bangla to English to confirm the reliability of the intended content and to avoid any vague and ambiguous language. The modified KAP survey was then piloted with 30 barbers, who did not take part in the final survey, to ensure content clarity and integrity, and to remove redundancy. The final version of the KAP survey used in this study consisted of 31 items, including 17 questions regarding knowledge about HBV and HCV, 7 questions regarding attitudes toward HBV and HCV, and 7 questions regarding practices toward HBV and HCV.

### 2.3 Sample size calculation

Sample size was calculated using the following formula

$$n\;=\;\frac{Z^{2}p\;\times \;q}{e^{2}}$$

z ​= ​1.96 (95% CI).

p ​= ​0.5 (Assuming prevalence of 50% of subjects having an inadequate knowledge and practice).

q ​= ​1-p (0.5).

e ​= ​acceptable error (10% of p value) ​= ​0.050

n ​= ​384.

### 2.4 Data collection

For the study purpose, Rajshahi city was divided into 20 zones based on markets where barbers’ shops were located. The number and location of barbershops in each zone were collected from the local people. On average, 2 to 3 barbers worked in a barbershop. It was assumed that eight barbershops from each zone selected by the random sampling method by lottery would be adequate to fulfill the sample size. Interviewers with educational status above higher secondary school (Grade 12) were trained for five days before data collection. Interviewers visited the selected barber shops and requested barbers to participate in the study. Face-to-face interviews were conducted with participating barbers. Each interview took an average of 30 ​min. The barbers were also observed during barbering 2–3 clients per shop for assessing instrument use practices. Before starting questions, the purpose of the study was discussed with the participants. Barbers unwilling to participate were excluded from the study. All participants provided written informed consent before enrollment. The study was approved by the Ethical Review Committee of Rajshahi Medical College, Bangladesh (Ref.RMC/ERC/2020/236/227).

### 2.5 Statistical analyses

All statistical analyses were performed in SPSS 20. Categorical variables were presented as counts and percentages, and continuous variables were presented as means (±standard deviations) or median. An individual knowledge score was calculated for each participant. Participants were given one point for each correct answer to the knowledge-related questions, with a minimum possible score of 0 and a maximum possible score of 17. Wrong answers or responses of “do not know” did not receive any points. A higher knowledge score indicated better knowledge about modes of transmission, complications, and prevention of HBV and HCV. Associations between knowledge score and various socio-demographic characteristics (age, monthly income, marital status and educational level) were assessed by Welch’s *t*-test. All analyses were performed at a 5% threshold for statistical significance.

## 3 Results

### 3.1 Socio-demographic profile of participants

A total of 403 barbers (mean age 29.2 ​± ​7.4 years) were included in the study. All the participants in the study were male. Table 1 showed the demographic characteristics of the participant. The majority of barbers (57.5%) were either illiterate (did not attend school) or had education up to primary school. More than one-third of the participants had income 10000 Bangladeshi Taka and below.

**Table 1:** Socio-demographic characteristics of barbers (n ​= ​403).

Participant characteristics	Results
Mean age (y)	27.1 ​± ​9.3
Education, n (%) Illiterate Primary Primary to SSC Above SSC	49 (12.1%) 183 (45.4%) 161 (40%) 10 (2.5%)
Monthly income, n (%) <10000 BDT (120 USD) 10000-20000 BDT (120–240 USD) >20000 BDT (240USD)	151 (37.4%) 212 (52.6%) 40 (10%)
Marital status, n (%) Single Married	200 (49.6%) 203 (50.4)

BDT, Bangladeshi Taka.

USD, US Dollar.

### 3.2 Knowledge about HBV and HCV

**Fig. 1:**
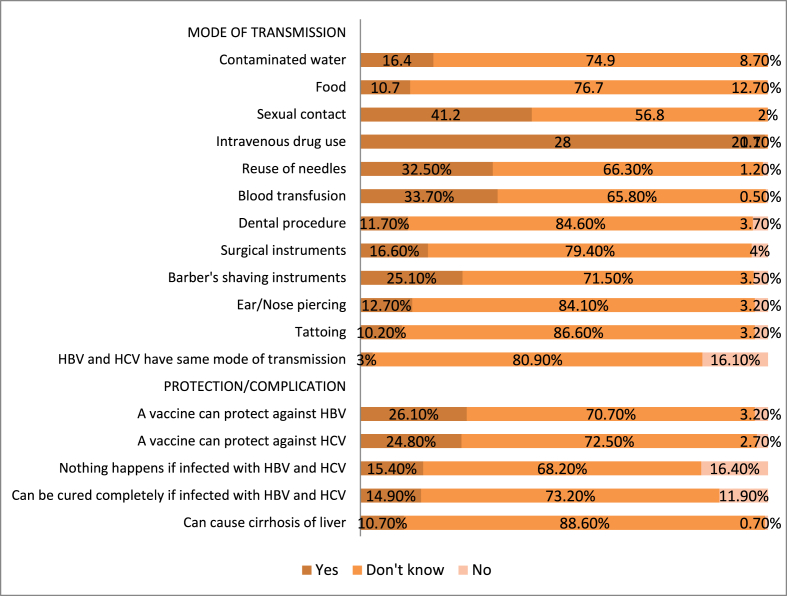
Responses to questions regarding knowledge of hepatitis B and C virus transmission, protection against the viruses and complications of hepatitis B and C viral infection among barbers (n ​= ​403).

From Fig. 1, it can be seen that most of the barbers had insufficient knowledge regarding modes of transmission and the different sources/risk factors of HBV and HCV. 41.2% of barbers knew that HBV and HCV could be transmitted through sexual route, and 33.7% knew that the viruses could be transmitted through blood transfusion. Only 25.1% of barbers knew that the viruses could be transmitted through their shaving instruments. Regarding protection against HBV, only 26.1% knew that there is a vaccine that can protect against HBV. A small percentage of participants (43,10.7%) knew that HBV and HCV could cause liver cirrhosis. Table 2 presents the associations between participant socio-demographic characteristics and knowledge scores. A positive association was seen between higher knowledge scores and postprimary education (P ​= ​0.011).

**Table 2:** Association between sociodemographic characteristics and knowledge of barbers about hepatitis B and C virus (n ​= ​403).

Participant characteristic	Knowledge score,mean	*P*
Age^a^ Age<32years Age≥32years	2.7 3.1	0.421
Education^a^ Primary or below Post-primary	2.4 3.4	0.011
Monthly income^a^ Income <12000 BDT (142 USD) Income ≥12000 BDT (142 USD)	2.6 3.3	0.099
Marital status Single Married	2.6 3.0	0.288

BDT, Bangladeshi Taka.

USD, US Dollar.

a Median is taken as cut off value.

### 3.3 Attitudes toward HBV and HCV

Table 3 described the attitudes of barbers toward HBV and HCV. One-third of the barbers (127, 31.5%) had not seen any information or radio/television programs on these diseases. Only 12.2% of barbers reported that they had been vaccinated against the HBV virus. Most of the participants (318, 78.9%) agree that it was essential to have periodic screening tests for HBV and HCV. 10.4% reported a history of blood transfusion, and 20.8% had a surgical operation.

**Table 3:** Responses to questions regarding attitudes of barbers toward Hepatitis B and C virus (n ​= ​403).

Attitude item	Yes, n (%)	No, n (%)
Agree to be personally tested for these infections	318 (78.9%)	85 (21.1%)
Is vaccinated against hepatitis B virus	49 (12.2%)	354 (87.8%)
Is currently substance abuser	7 (1.7%)	396 (98.3%)
Has had blood transfusion	42 (10.4%)	361 (89.6%)
Has had surgical operation/stitching	84 (20.8%)	319 (79.2%)
Has had tatoos	18 (4.5%)	385 (95.5%)
Has had body piercing	47 (11.7%)	356 (88.3%)

### 3.4 Practices toward HBV and HCV

Regarding barber’s practice, most barbers washed their hands before attending to each client (376, 93.3%). 57.1% of barbers cleaned instruments with disinfectant between clients; however, 88.7% washed their instruments with tap water after shaving the clients. 99.5% used a new blade on new clients, and most of them (371, 92.1%) disposed of the used blades into the regular garbage system. 98.8% of barbers used antiseptic on skin cut (Table 4).

**Table 4:** Barbers practices toward Hepatitis B and C virus (n ​= ​403).

Practices items	Yes, n (%)	No, n (%)
Washes hands before each client	376 (93.3%)	27 (6.7%)
Cleans instruments with disinfectants between clients	230 (57.1%)	173 (42.9%)
Washes instruments with tap water after shaving	387 (96%)	16 (4%)
Clean instruments after shaving	401 (99.5%)	02 (0.5%)
Uses new blade on new client	401 (99.5%)	02 (0.5%)
Uses anticeptic on skin cut	398 (98.8%)	05 (1.2%)
Disposes used blades in the garbage	371 (92.1)	32 (7.9)

## 4 Discussion

Studies conducted in the past three decades have shown a fluctuating prevalence of HBV and HCV among different population groups in Bangladesh who are mostly asymptomatic [31,32]. This indicates persistence of the source of infection in the community. Many of the studies have found hair cutting and shaving by barbers to be one of the major risk factors for the transmission of HBV and HCV infection. Poor knowledge regarding HBV and HCV transmission, lack of awareness regarding prevention and complication of the infection, and lack of safe practice by barbers could be the major causes of transmission of infection from barber’s shop. This current study had investigated the knowledge, attitudes, and practices of barbers about hepatitis B and C virus and showed poor knowledge of the barbers regarding modes of transmission, complications, and prevention of the viruses. The study also revealed a moderate level of attitudes and relatively good practices of the participants towards HBV and HCV to prevent their transmission.

In this study, all the respondents were male. In Bangladesh, males are usually involved in barbering, whereas beauty parlor workers are usually female. The correct response to the questions regarding modes of transmission of HBV and HCV ranging from 3% to 41.2%. Only 25.2% of the barbers knew that the viruses could be transmitted through their shaving instruments. The majority of the participants didn’t know the consequences and complications of these viral infections. 26.1% of the barbers knew that HBV infection could be prevented by vaccination. The findings of our study are generally consistent with those of the former study reports from Pakistan, Egypt, and Yemen, but a relatively lower level of knowledge and awareness of the barbers about HBV and HCV in our study is noticed in comparison to other similar studies across the world [[26], [27], [28],^30^,^33^]. This may be due to the lower educational level of the participants in this study than those of the other studies. Most of our study participants had an educational level below secondary school. A positive association between low educational level and poor knowledge about HBV and HCV among barbers had also been described in our study.

About 79% of barbers in this study agreed to be personally tested for HBV and HCV infections and only 12.2% of the barbers were vaccinated against HBV. A lower vaccination rate has also been noticed among barbers in other previous studies [26,27,33]. 10.4%, 20.8%, and 11.7% of the study participants had a history of blood transfusion, surgical operation/stitching, and body piercing respectively. Lack of vaccination and the presence of risk factors for transmission made the barbers vulnerable to the HBV and HCV infection acquired by accidental exposure to the blood and bodily fluids of their customers during shaving and haircutting. Though the hepatitis B vaccine is included in the Expanded Program of Immunization (EPI) in 2003 in Bangladesh with coverage exceeds 90% of infants [34], adults remain at risk for both hepatitis B and C virus. A recent study by Modhusudon et al. found that 64.7% of the nonvaccinated hospital outdoor patients were tested positive for anti-HBc, indicating that they had been exposed to the hepatitis B virus (HBV) at least once in their lifetime. Exposure to HBV (anti-HBc positive) was higher among low-income people, saloon users, and those older than 30 years of age [35].

A large proportion of the barbers (42.9%) did not clean their instruments with disinfectants and some of them even did not clean the instruments at all. 99.5% of the participants used a new blade on a new client and most of them (92.1%) disposed of their used blades into the garbage. Our study findings showed relatively good practices among barbers in comparison to other similar studies. One of the explanations of the poor knowledge and relatively good practice among the barbers in this study is that the barbers might have been aware of being observed, and may, therefore, have modified their practices. Another explanation could be that they were doing the practices for preventing other diseases having the same modes of transmission such as HIV/AIDS because overall a relatively higher level of knowledge about HIV/AIDS is noticed among urban and rural men in Bangladesh [36]. Besides, it may be possible that about 8% of the barbers who do not dispose of blades in the bin, may have been intending to reuse them.

This current study had some limitations. First, there are no existing Bangla validated KAP surveys regarding HBV and HCV. This study utilized a survey adapted from KAP surveys conducted in other settings and other languages. Therefore, the actual psychometric properties of the tool used in this investigation are unknown. Secondly, like other KAP surveys, this study may be subjected to social desirability bias. This may have resulted in an over-reporting of the good practices, for example, the proportion of the participants disposed of their used blades into bins. Finally, the participants in this study may not be representative of the barber community outside Rajshahi city of Bangladesh. Thus, the findings of this study may not be applicable to the barbers in other parts of the country.

In conclusion, Barbers’ knowledge level about HBV and HCV is very poor in the north-west part of Bangladesh. The majority of the barbers are not vaccinated against HBV. Many of them practiced unsafely during barbering. Specific measures should be taken to increase awareness of the barbers regarding modes of transmission, complications, and prevention of HBV and HCV. Measures should also be taken to bring the barber under the coverage of vaccination against HBV. Concerned authorities should provide adequate training to the barbers to increase the safe practice of these workers on these health hazards. Further studies should be done in other parts of the country to reveal the total picture of the country regarding knowledge, attitude, and practice of barbers about HBV and HCV.

## Ethical approval

All participants provided written informed consent before enrollment. The study was approved by the Ethical Review Committee of Rajshahi Medical College, Bangladesh (Ref.RMC/ERC/2020/236/227).

## Competing interests

The authors declare that they have no known competing financial interests or personal relationships that could have appeared to influence the work reported in this paper.

## Funding

This research did not receive any specific grant from funding agencies in the public, commercial, or not-for-profit sectors.
